# Bioinformatic analysis identifies key transcriptome signatures in temporal lobe epilepsy

**DOI:** 10.1111/cns.13470

**Published:** 2020-11-22

**Authors:** Qing‐Lan Chen, Lu Xia, Shao‐Ping Zhong, Qiang Wang, Jing Ding, Xin Wang

**Affiliations:** ^1^ Department of Neurology Zhongshan Hospital Fudan University Shanghai China; ^2^ CAS Center for Excellence in Brain Science and Intelligence Technology Shanghai China; ^3^ Department of The State Key Laboratory of Medical Neurobiology The Institutes of Brain Science and the Collaborative Innovation Center for Brain Science Fudan University Shanghai China

**Keywords:** epileptogenesis, microarray, robust rank aggregation, temporal lobe epilepsy

## Abstract

**Aims:**

To identify transcriptome signatures underlying epileptogenesis in temporal lobe epilepsy (TLE).

**Methods:**

Robust rank aggregation analysis was used to integrate multiple microarrays in rodent models of TLE and determine differentially expressed genes (DEGs) in acute, latent, and chronic stages. Functional annotation and protein‐protein interaction analysis were performed to explore the potential functions of the DEGs and identify hub genes with the highest intramodular connectivity. The association between hub genes and hippocampal sclerosis/seizure frequency was analyzed using publicly available RNA‐sequencing datasets from TLE patients. We subsequently established a pilocarpine‐induced status epilepticus (SE) model in rats and validated mRNA expression of hub genes by quantitative reverse transcription PCR (qRT‐PCR).

**Results:**

The DEGs in the acute, latent, and chronic phases of TLE in animal models were prominently enriched in inflammatory response. Hub genes identified in the acute phase mainly participated in biological processes including inflammation, blood‐brain barrier damage, and cell adhesion. The hub genes in the latent phase were related to microglia/macrophage activation (*Emr1* and *Aif1*) and phagocytosis (*Cd68, Tyrobp,* and *Lyz*). In the chronic phase, the hub genes were associated with activation of complements and microglia/macrophages. We further found that some hub genes identified in human TLE, such as *Tlr2, Lgals3,* and *Stat3,* were positively correlated with seizure frequency. Other hub genes, including *Lgals3* and *Serpine1,* were associated with hippocampus sclerosis. qRT‐PCR analysis confirmed that the mRNA levels of hub genes in rat hippocampus were significantly up‐regulated after SE induction.

**Conclusions:**

Our integrated analysis identified hub genes in different stages of epilepsy. The functional annotations suggest that the activation and phagocytic activities of microglia/macrophages may play critical roles in epileptogenesis of TLE.

## Graphical abstract

Activated phagocytic microglia may regulate the formation of abnormal neural circuits in temporal lobe epilepsy (TLE). After an epileptogenic injury, microglia in hippocampus was activated with up‐regulated lysosomal molecules including CD68, LAMP1, LYZ, and LAPTM5. The expressions of phagocytosis‐related molecules like C1QA, C1QB, C3, and TYROBP were also up‐regulated. Activated phagocytic microglia could engulf apoptosis neurons, newborn neurons, and synapse with the help of complement and TYROBP, thus regulating the abnormal neural circuits and epileptogenesis in TLE. In chronic stage, the occurrence of spontaneous seizures further activated microglia and complements and exacerbated abnormal neuronal circuits.

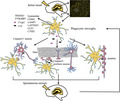

## 1 INTRODUCTION

Temporal lobe epilepsy (TLE) is characterized by refractory seizures, significant cognitive decline, and depression. TLE accounts for 36% of intractable epilepsy.^1^ Epileptogenesis is a progressive process in which an insult to a normal brain culminates in the occurrence of spontaneous seizures. There are three stages in epileptogenesis—the acute, latent, and chronic stages. Pathophysiologic changes occur in the injured zone in the acute phase, which is followed by the gradual maturation of epileptic circuit in the latent phase and the ultimate appearance of spontaneous recurrent seizure (SRS) in the chronic phase.^2^ Animal models that mimic the clinical and histopathological features of TLE are particularly helpful to study molecular mechanisms in different stages of epileptogenesis.^3^ Previous studies identified several pathological mechanisms of epileptogenesis involving immune responses, synaptic plasticity, neurodegeneration, neurogenesis, alterations in metabolism, and blood‐brain barrier (BBB) integrity.^2^, ^4^ Alterations of transcriptomic profiles have been reported in brains from epilepsy patients and animal models.^5^ Approximately 2000 genes were published to be differentially expressed in TLE.^6^ Characterizing stage‐specific gene expression profiles and identifying key regulatory genes are crucial to elucidate the molecular mechanisms of epileptogenesis in TLE.

High‐throughput microarray and RNA‐sequencing (RNAseq) analyses provide superior platforms for a comprehensive and unbiased transcriptome‐wide analysis and have been widely applied in epileptic studies in animal models and in patients in the past two decades. However, concerns about representativeness and repeatability of an individual microarray/RNAseq study have been raised.^7^ The heterogeneities in experimental design, animal model, sampling time, and sample size confounded result interpretation.^7^ Wang et al compared the lists of differentially expressed genes (DEGs) from different microarray studies in TLE and found that only 53 among over 2000 DEGs altered in more than two studies.^6^ An integrated analysis could efficiently overcome the low validity and reproducibility of an individual experiment, and may help to identify candidate genes that are implicated across different studies. Several bioinformatic analyses on microarray studies in epilepsy have been published recently. However, three analyses were based on summarizing published results rather than performing integrated analysis of original microarrays.^5^, ^7^, ^8^ Another study integrated multiple rodent TLE microarrays with RankProd package, whereas their research of key gene regulators was derived from one dataset instead of from gene expression profiles across different microarrays.^9^

Robust rank aggregation (RRA) algorithm has been developed for microarray integration in 2012. The basis of RRA is to compare the actual list of each dataset with a null assumption of random order and to screen the DEGs by assigning a significance score for each gene. The aggregated list kept only the statistically relevant DEGs.^10^ RRA has been used for integrated microarray analysis of triple‐negative breast cancer and discovered key dysregulated miRNAs.^11^ Song et al also identified hub genes in prostate cancer using RRA.^12^ Moreover, RRA can process the variable gene lists and handle missing ranks from different microarray platforms.^10^

In the current study, we conducted an integrated analysis on TLE microarrays using RRA to identify key genes consistently expressed across different epileptic models and platforms. Further gene annotation and protein interaction analysis identified key pathways and hub genes in three phases of epilepsy. The association between hub genes and hippocampal sclerosis (HS)/seizure frequency was also analyzed using RNA sequencing datasets of TLE patients. In addition, the expression of several hub genes was validated in a rat pilocarpine model of TLE using quantitative reverse transcription polymerase chain reaction (qRT‐PCR).

## 2 MATERIALS AND METHODS

### 2.1 Microarray datasets of epilepsy

The microarray datasets were obtained through searching the Gene Expression Omnibus (GEO) database. We systematically searched the microarray studies using the terms: “epilepsy” or “seizure” or “pilocarpine” or “kainate” or “kainic acid” or “electrical stimulation”. Datasets or samples were included according to the following criteria: (1) published after year 2009, (2) including both experimental groups and controls, (3) raw data or series matrix files are available in GEO, and (4) the animal model was induced by either pilocarpine or kainic acid or electrical stimulation. Microarrays were excluded if animals have been treated with interventions unrelated to the establishment of the epilepsy model.

### 2.2 Dataset preprocessing and obtaining DEGs

We downloaded raw data or series matrix files of six microarray datasets from GEO database. In order to reduce heterogeneity of analysis, only the ipsilateral hippocampus samples in each microarray were included. The corresponding annotation documents were used to transform the probes into gene symbols. If multiple probes matched the same symbol, the mean signal intensity was calculated. Each dataset was normalized by RMA algorithm. Since gene expression patterns may change with the development of epilepsy, we divided our analysis into three distinct phases: acute stage (0‐3d), latent stage (4‐14d), and chronic stage (the first SRS or after 14d). The samples in six included microarrays were regrouped according to their sampling time after model establishment. The common control was used if there was no control for each epileptic group in the microarray. The DEGs between epileptic and control hippocampus in different phases were identified using the “limma” package in R language. The cutoff criteria were set as |log_2_ fold change (FC)| >1 and adjusted *P*‐value <0.05.

### 2.3 RRA analysis to integrate microarrays in different phases

To minimize the deviation caused by the analysis of a single array, we integrated the results of several microarrays by adopting RRA algorithm.^10^ Before RRA analysis, up‐ranked and down‐ranked matrixes of all genes in each dataset were generated based on log_2_ FC between epileptic and control groups. The log_2_ FC matrixes were then merged to get a combined matrix. “RobustRankAggreg” package was applied to integrate the combined log_2_ FC matrix. The adjusted *P*‐value indicated the gene ranking in the final list. Genes with Bonferroni‐adjusted *P*‐value <0.05 and the |log_2_ FC|>0.5 were considered as robust DEGs.

### 2.4 Pathway and process enrichment analysis

The DEGs obtained in RRA analysis were uploaded to Metascape (http://metascape.org/gp/index.html) to perform pathway and process enrichment analysis.^13^ Terms with a *P*‐value <0.01, a minimum count of 3, and an enrichment factor >1.5 were collected.

### 2.5 Protein‐protein interaction (PPI) analysis and identification of hub genes

Hub genes are defined as genes of high connectivity with other genes. The alteration of hub genes will cause prominent changes in the gene network. The DEGs obtained in RRA analysis were uploaded to STRING database to predict the interactions among proteins.^14^ The minimum required interaction score was set as 0.4. Cytoscape software 3.6.1 was used to visualize the protein networks. The top 5 connective genes in each phase were identified as hub genes.

### 2.6 RNA‐SEQUENCING DATA ANALYSIS IN BRAINS FROM HUMAN TLE PATIENTS

The raw count matrices of the two RNA sequencing datasets (GSE127871 and GSE71058) were downloaded from the GEO database. In GSE127871, the hippocampal tissues resected from 12 intractable TLE patients with seizure frequencies ranging from 0.33 to 120 seizures per month were sequenced. The CPM of genes was calculated using the cpm function of the “edgeR” package. A Spearman rank correlation analysis was conducted using SPSS 24 to assess the association between cpm of hub genes and seizure frequency. Genes with coefficient *P*‐value <0.05 were considered to be significantly associated with seizure frequency. In GSE71058, the dentate gyrus of 5 TLE patients with HS and 7 without HS were sequenced. The DEGs were obtained using “DESeq2” package. Genes with the |log_2_ FC| >1 and adjusted *P*‐value <0.05 were considered as HS‐related genes. The scatterplots and barplots were prepared by GraphPad Prism 7 software.

### 2.7 Animal model

Male adult Sprague Dawley rats (6‐8 weeks, 200‐250 g) supplied by Shanghai Charles River Laboratory were used in this study. The rats were housed four per cage under controlled temperature (22‐25°C) and humidity (50%‐60%) with 12‐hour light/dark cycle. Rats had free access to water and food. All experimental procedures were approved by the Animal Ethics Committee of Zhongshan Hospital.

The animals were randomly divided into experimental and control groups. Rats received intraperitoneal (i.p.) injection of lithium chloride (LiCl, 127 mg/kg; Sigma, St. Louis, MO, USA), followed by scopolamine hydrobromide (1 mg/kg, i.p., Harvest Pharmaceutical Co. Ltd, China) 24 hours after LiCl. Pilocarpine (45 mg/kg i.p.; Sigma‐Aldrich) was given 30 minutes later to induce seizure. The control animals received equal amounts of normal saline. The pilocarpine‐induced seizures were evaluated according to a modified Racine scale.^15^ Animals with seizure scale greater than or equal to Racine stage 4 and lasted for 30 minutes (status epilepticus, SE) were included. All animals received diazepam injection (10 mg/kg, i.p., Tianjin, China) 30 minutes after the onset of SE to terminate seizures. The experimental animals were sacrificed 48h (n = 4), 7d (n = 4), or 35d (n = 3) after SE. The control animals (n = 4) were sacrificed 7d after SE.

### 2.8 RNA extraction and qRT‐PCR

Total RNA was extracted from hippocampus of rats using TRIzol (Invitrogen, CA, USA) according to the manufacturer's instructions. cDNA synthesis was performed using the ABScript II reverse transcription premixed solution (Abclonal, Wuhan, China). In order to quantify the transcripts of interest genes, qRT‐PCR was performed using a SYBR Green Master Mix (Yeasen, Shanghai, China) on LightCycler 480 (Roche, Switzerland). The target mRNA levels were normalized to GAPDH internal control gene. Expression data were analyzed according to the 2‐△△Ct method.^16^ Primers are listed in Table S1.

### 2.9 Statistical analysis

For qRT‐PCR analysis, ANOVA was used if data were normal distribution and homogeneous variance. Otherwise, the Kruskal‐Wallis test was used. An adjusted *P‐*value <0.05 was considered to be statistically significant. The data were reported as mean ± standard error. The GraphPad Prism 7 software was used for data analysis and plotting.

## 3 RESULTS

### 3.1 Information of included microarrays

According to the inclusion criteria, GSE14763, GSE49849, GSE73878, GSE47752, GSE49030, and GSE88992 were included in this study. The detailed information of these datasets is shown in Table 1. We divided our analysis into the acute, latent, and chronic stages according to the duration after model establishment. The acute group included GSE49030, GSE88992, GSE14763, and GSE47752. The latent group included GSE14763, GSE49849, GSE73878, and GSE47752. The chronic group included GSE14763, GSE49849, and GSE73878. The samples in each stage are listed in Table 1.

**TABLE 1:** Characteristics of the six microarray datasets in our integrated analysis

GSE ID	Control	Acute stage (0‐3d)	Latent stage (4‐14d)	Chronic stage (14d later)	Tissue	Platform	Year
GSE73878	IL^a^‐sham‐7d: 4 IL‐sham‐28d: 5	N/A	IL‐KA^b^‐7d: 8	IL‐KA‐28d: 9 IL‐KA‐60d:14	M^e^‐H	GPL6885	2015
GSE49849	Sham‐7d: 5sham‐30d: 5	N/A	stimu^c^‐7d: 5	stimu‐30d: 5	R^h^‐DG	GPL11534	2013
GSE88992	Saline‐6h: 3 Saline‐12h: 3 Saline‐24h: 3	KA‐6h: 3 KA‐12h: 3 KA‐24h: 2	N/A	N/A	M‐H^g^	GPL1261	2016
GSE14763	Control: 3	Pilo^d^‐3d: 5	Pilo‐7d: 5	Pilo‐chronic: 5	R‐H	GPL2896	2009
GSE47752	Pilo control: 17 KA control: 12	Pilo‐1d: 12 KA‐1d: 12 Pilo‐3d: 12 KA‐3d: 13	Pilo‐10d: 12 KA‐10d: 12	N/A	R‐DG^f^	GPL1355	2013
GSE49030	Control‐1h: 9	KA‐1h: 3 KA‐4h: 6 KA‐8h: 3 KA‐24h: 3	N/A	N/A	M‐H	GPL1261	2013

Note

A total of six microarrays were included in the integrated analysis. In these microarrays, seizure was induced in rat or mouse by either pilocarpine or kainic acid or electrical stimulation. Hippocampus or dentate gyrus of the epileptic rats or mice was sampled at different time points for expression profile detection in microarray platform. The samples in six included microarrays were divided according to sampling time after model establishment into three groups: acute stage (0‐3d), latent stage (4d‐14d), and chronic stage (the first spontaneous recurrent seizure or after 14d).

^a^ ipsilateral;

^b^ kainic acid;

^c^ electrical stimulation of amygdala;

^d^ pilocarpine;

^e^ mouse;

^f^ dentate gyrus;

^g^ hippocampus;

^h^ rat.

### 3.2 Identification of DEGs in epilepsy models

**FIGURE 1:**
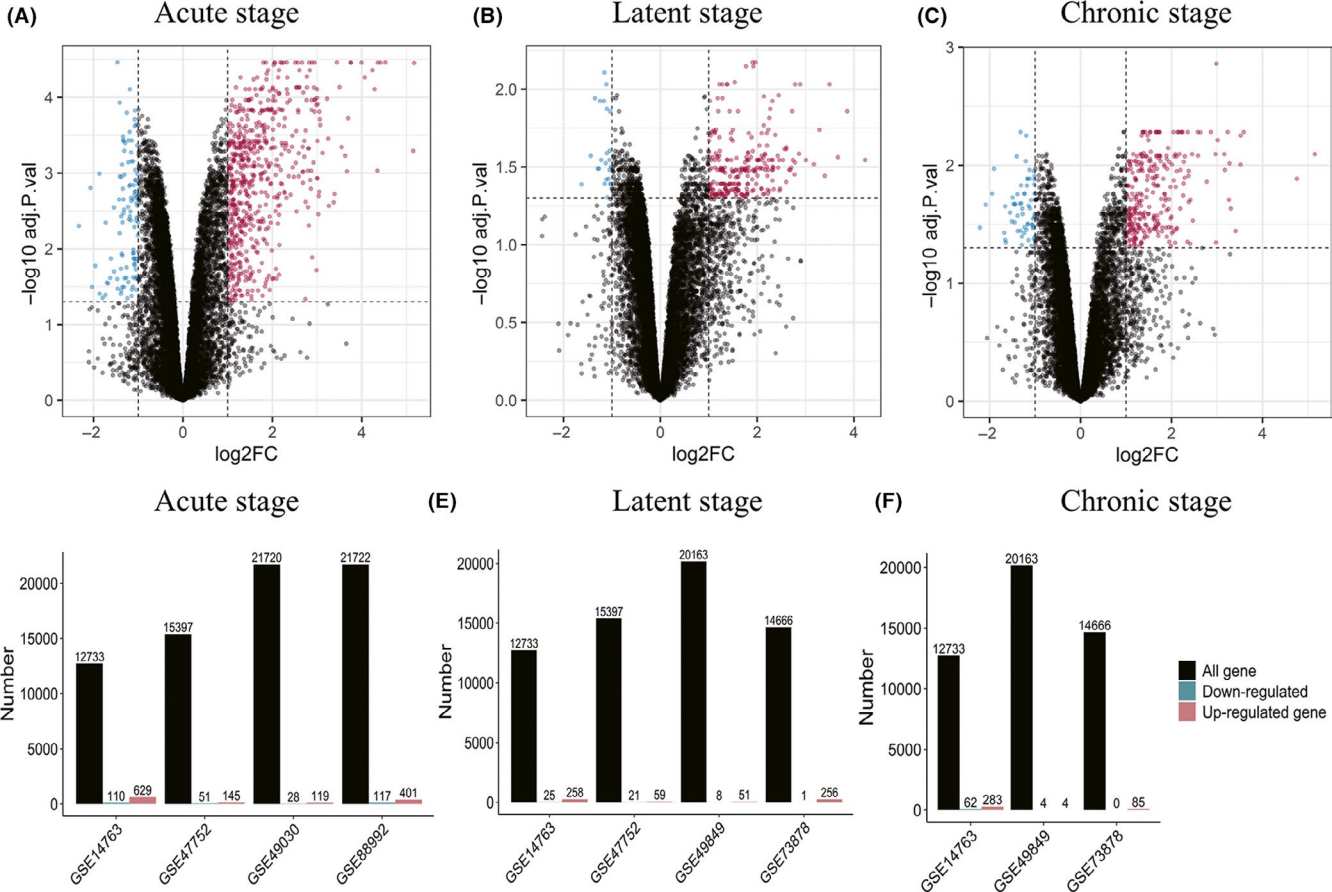
Gene expression distributions of microarrays in three stages of epilepsy. (A‐C): Representative volcano plots of the microarrays in the acute, latent, and chronic stages (GSE14763). Red points represented up‐regulated genes, while blue points represented down‐regulated genes. Black points indicate genes with no significant difference. (D‐F): Barplots of gene expression distributions of microarrays in the acute, latent, and chronic stages. Numbers of total genes, up‐regulated genes, and down‐regulated genes of each microarray were labeled at the top of the barplots

We standardized each microarray dataset using RMA algorithm to achieve homogeneity between samples. The boxplots after standardization are shown in Figure S1. The “limma” package in R software was used to screen the DEGs according to the cutoff criteria. The volcano plots and barplots demonstrated the gene expression in the acute (Figure 1A,D), latent (Figure 1B,E), and chronic stages (Figure 1C,F) of TLE. The number of DEGs obtained in each microarray varies greatly, ranging from over 100 to more than 700 in acute stage, which may be caused by differences in modeling, species, detection platform, severity, and duration of SE.

### 3.3 RRA integrated analysis

**FIGURE 2:**
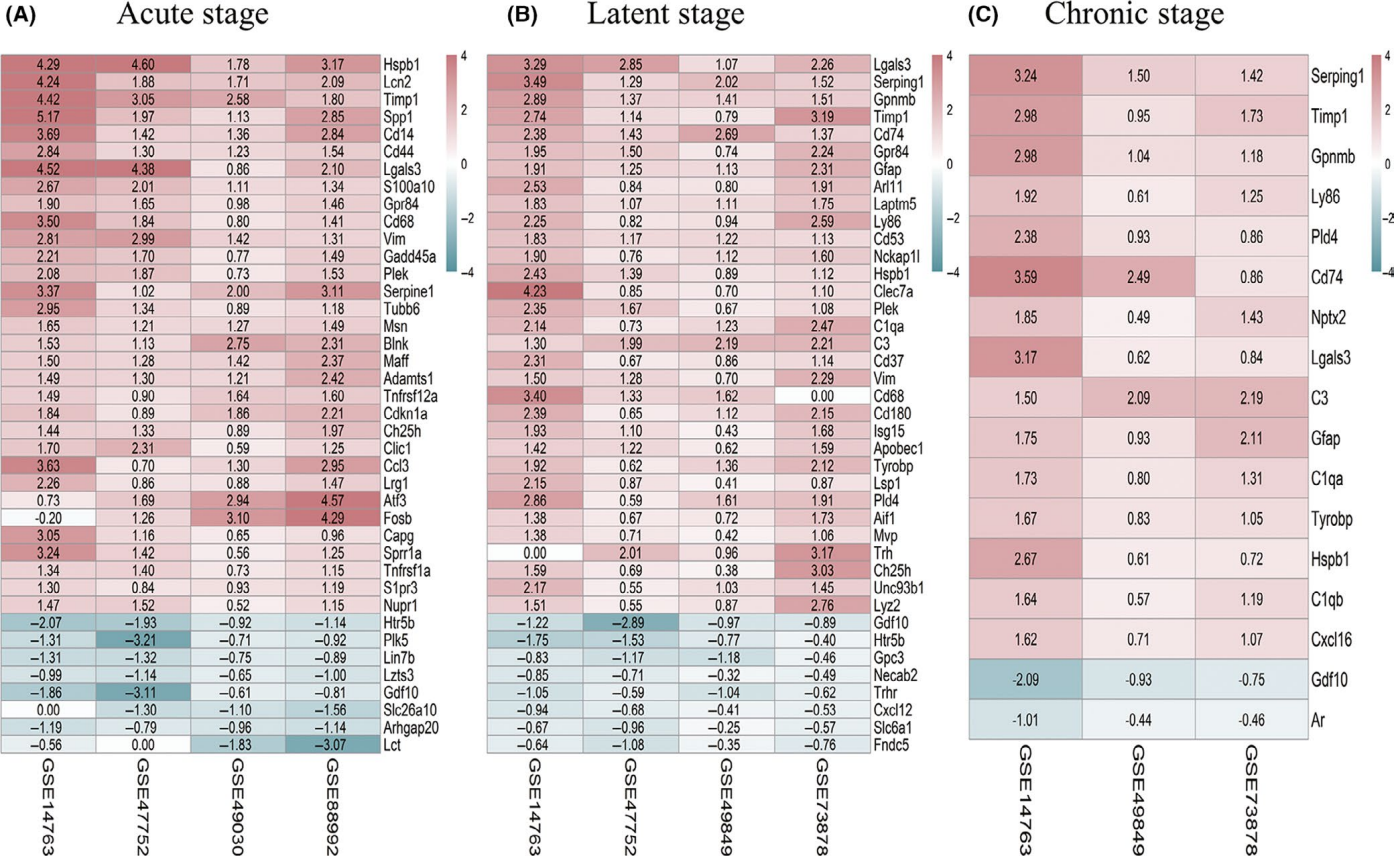
The robust differentially expressed genes (DEGs) obtained by integrating multiple microarrays using robust rank aggregation (RRA) algorithm in three stages of epilepsy. RRA algorithm assigned *P*‐value and log_2_ |fold change (FC)| to each gene and ranked the genes by the assigned *P*‐value. Genes with the Bonferroni‐adjusted *P‐*value <0.05 and |log_2_ FC|>0.5 were strictly kept in the final aggregated lists as the robust DEGs. (A‐C): Heat maps of the top up‐ and down‐regulated DEGs in the final aggregated lists identified by RRA analysis in the acute, latent, and chronic stages. The top 32 up‐regulated and 8 down‐regulated RRA genes in the acute and latent stages and all RRA genes in the chronic stage were displayed. Red represents up‐regulated genes, while blue represents down‐regulated genes. The depth of the color represents the size of the log_2_ FC

Through RRA integration, we were able to obtain the robust DEGs that were stably up‐regulated or down‐regulated across microarrays in each stage. We identified 60 robust DEGs in acute period (45 up‐regulated and 15 down‐regulated), 75 in latent period (66 up‐regulated and 9 down‐regulated), and 17 in chronic period (15 up‐regulated and 2 down‐regulated). The robust DEGs in each period are shown in Figure 2 and Table S2.

### 3.4 Pathway and process enrichment analysis

**FIGURE 3:**
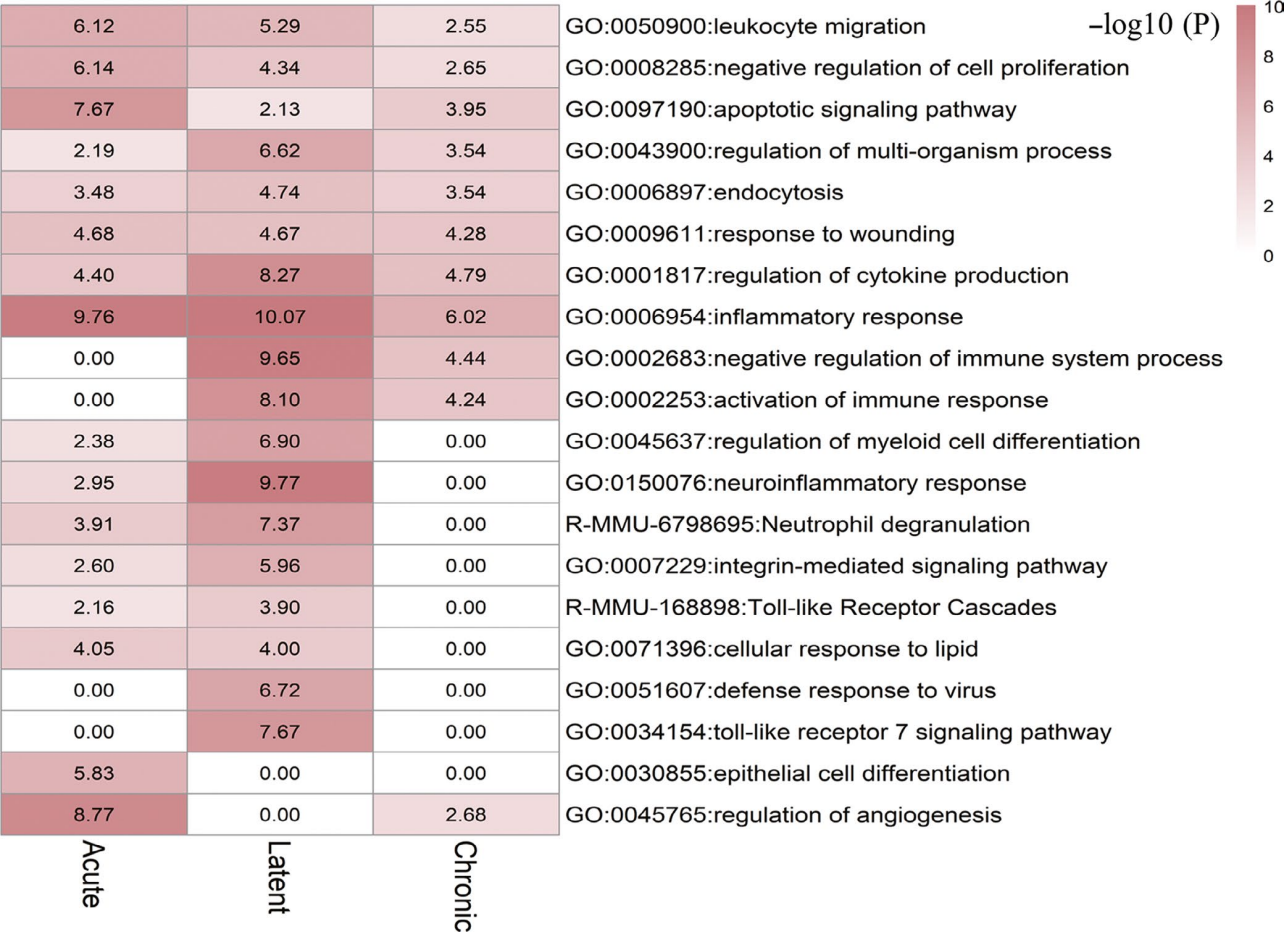
The representative heat map of pathway and process enrichment analysis identified by Metascape using robust DEGs obtained by RRA analysis in the acute, latent, and chronic stages. In Metascape, terms with a *P*‐value <0.01, a minimum count of 3, and an enrichment factor >1.5 are collected and grouped into clusters based on their membership similarities. A similarity >0.3 is considered a cluster. The top 20 clusters were chosen according to Log_10_(P) ranking and displayed. The color scale represents the value of −log_10_ (P). The items enriched are listed on the right of each row

The pathway and process enrichment analyses were then performed on the robust DEGs identified by RRA analysis in each stage (Figure 3, Table S3). A pronounced role of inflammation was exhibited persistently in all three phases of epilepsy, especially in the acute and latent stages. Biological processes including regulation of angiogenesis, apoptotic signaling pathway, endothelial cell differentiation, negative regulation of cell proliferation, and leukocyte migration were highly enriched in acute stage compared to the other two stages. In latent stage, biological processes such as neuroinflammatory response, Toll‐like receptor 7 signaling pathway, defense response to virus, negative regulation of immune system process, regulation of cytokine production, and integrin‐mediated signaling pathway were prominently enriched. Due to the small number of DEGs identified in the chronic stage of TLE, the chronic stage‐associated biological processes were relatively less compared to the acute and latent stages and mainly related to inflammation, immune responses, and apoptotic signaling pathway.

### 3.5 Protein‐protein interaction (PPI) network analysis and identification of hub genes

**FIGURE 4:**
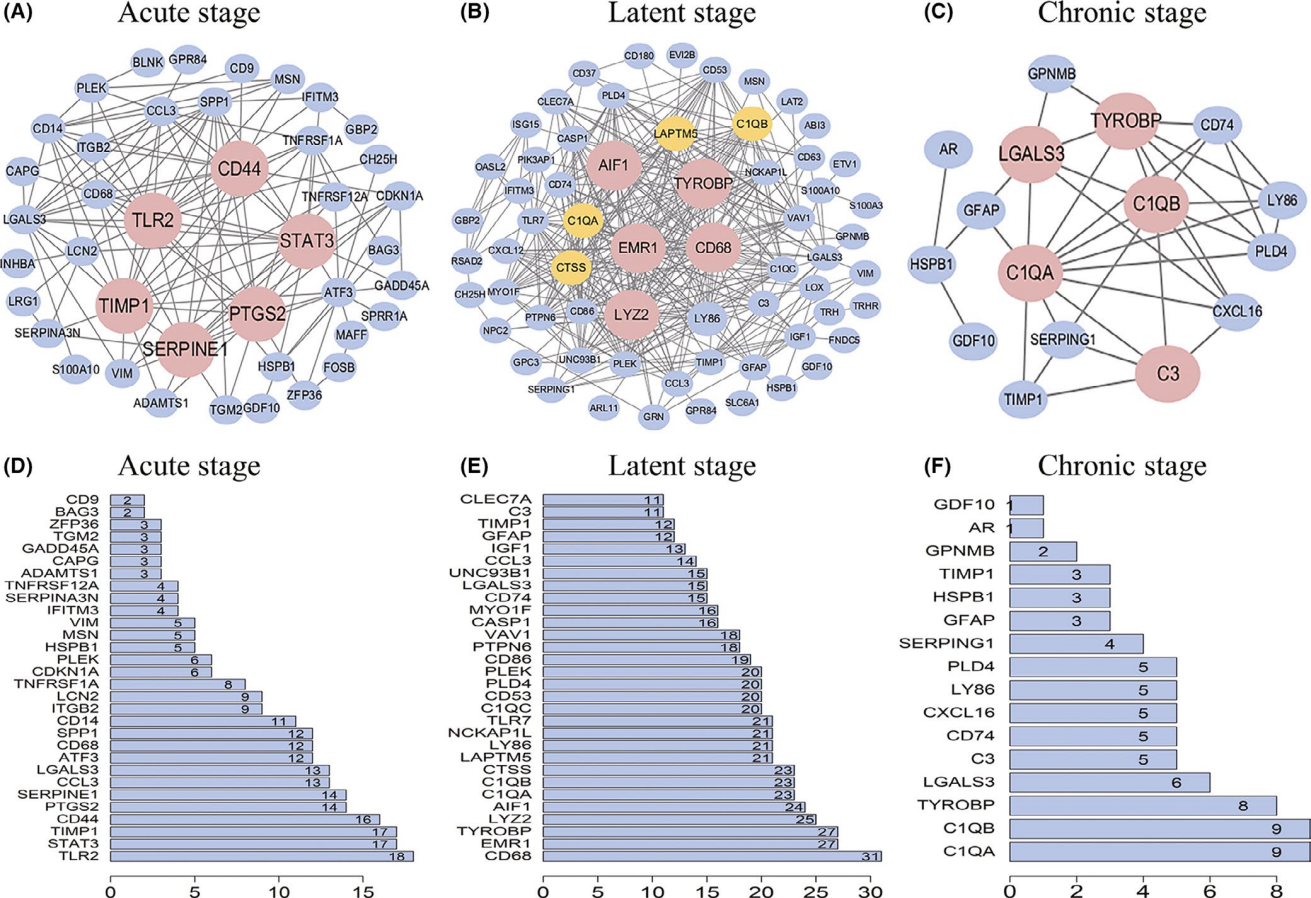
The protein‐protein interaction (PPI) network analysis was performed using STRING database of differentially expressed genes (DEGs) obtained by robust rank aggregation (RRA) analysis in the three stages of epilepsy. In the PPI network, each node represents a protein encoded by DEGs. The edge between nodes represents the interaction of the proteins. The edges of each RRA gene were counted and ranked. Hub genes were genes with top 5 connectivity. A‐C: PPI network of DEGs in the acute, latent, and chronic stages of epilepsy, respectively. Red indicates the hub genes with the top 5 connectivity. Yellow indicates several important RRA genes with connectivity second to hub genes. Blue represents other RRA genes. D‐F: The barplots of top 30 genes with most connections in the acute and latent stages, and all genes in the chronic stage of epilepsy, respectively. The number labeled at the right side of the barplots represents the edges counts of the DEGs

In the PPI analysis, the top 5 connective genes were identified as hub genes for their robust expression and high connectivity (Figure 4, Table S4). The hub genes in acute stage were *Tlr2, Stat3, Timp1, Cd44, and Ptgs2* (Figure 4A,D). The edges of *Serpine1* were the same as *Ptgs2;* thus, *Serpine1* was also considered as a hub gene in acute phase (Figure 4A). The protein interaction network in the latent stage was the most complicated among all stages (Figure 4B). The hub genes in latent stage were identified as *Cd68, Emr1, Tyrobp, Lyz2, and Aif1* (Figure 4B,E). The hub genes in chronic stage were *C1qa, C1qb, Tyrobp, Lgals3, and C3* (Figure 4C,F).

### 3.6 Hub genes associated with seizure frequency and hippocampal sclerosis in human TLE

**FIGURE 5:**
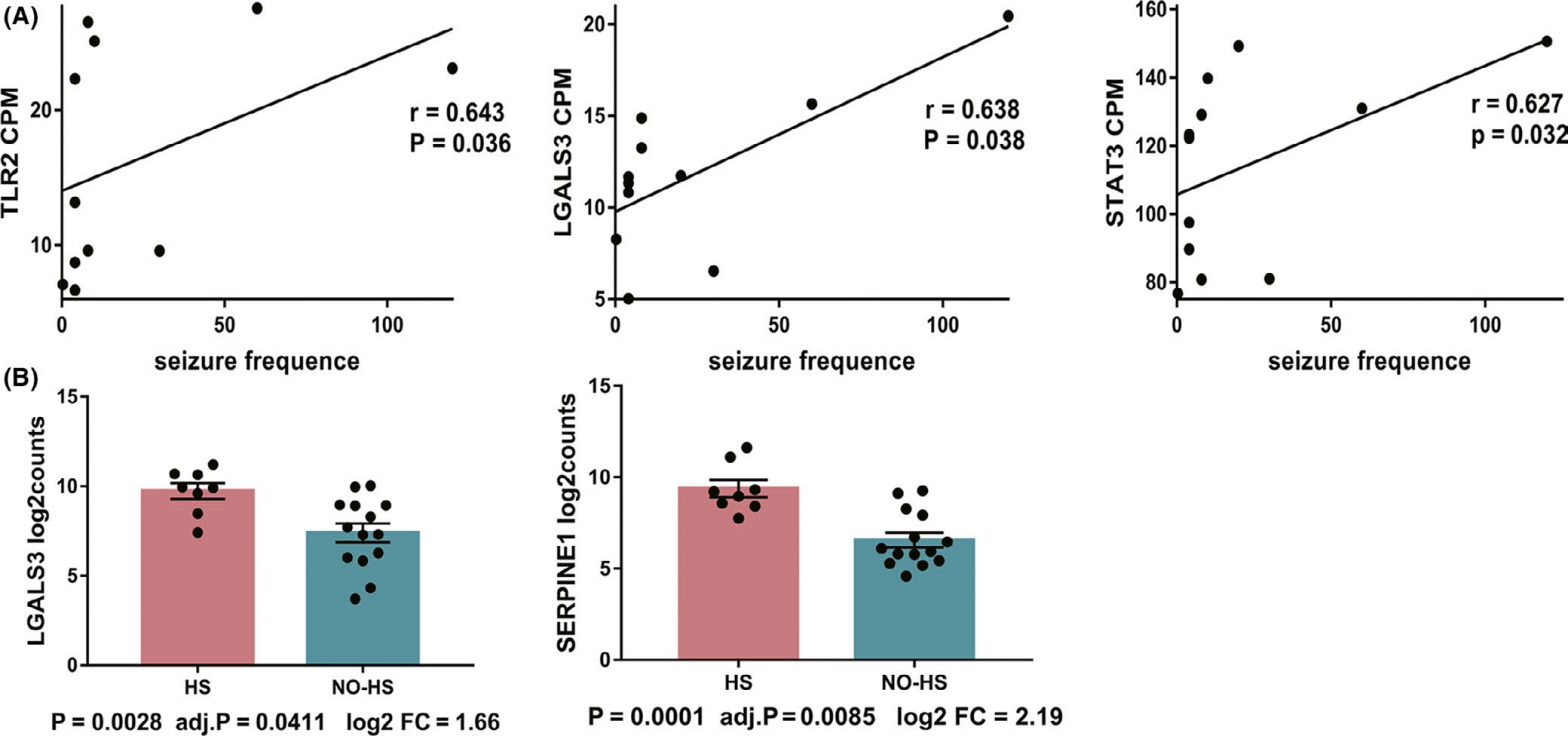
The hub genes associated with seizure frequency and hippocampal sclerosis in patients with temporal lobe epilepsy (TLE). A. Scatter plots showing the correlations between hub genes and seizure frequency in TLE patients in GSE127871. *r* = Spearman's rank correlation coefficient between the cpm of genes and seizure frequencies. The dark lines are the regression lines fitted by linear modeling. B. Barplots showing the differential expression of hub genes analyzed by DESeq2 package between the no‐HS and HS groups in TLE patients in GSE71058. Bottom of each plot showing the *P*‐values, FDR‐adjusted *P*‐value, and log_2_ (fold change (FC)) of genes

Among the abovementioned 16 hub genes in three stages of epileptogenesis (6 in the acute, 5 in the latent and 5 in the late stage), the hub genes *Tlr2, Lgals3,* and *Stat3* were positively correlated with seizure frequency in GSE127871 (Figure 5A). *Lgals3* and *Serpine1* were significantly increased in TLE patients with HS compared with those without HS (NO‐HS) in GSE71058 (Figure 5B).

### 3.7 The validation of hub genes in rat model of pilocarpine‐induced SE

**FIGURE 6:**
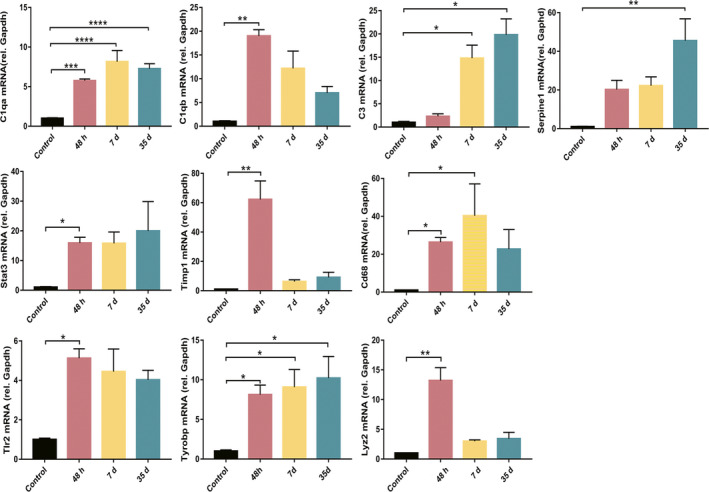
The expression of several hub genes was validated in the rat hippocampus by real‐time quantitative PCR at 48h (n = 4), 7d (n = 4), and 35d (n = 3) after pilocarpine‐induced status epilepticus. The control animals received equal amounts of vehicle (n = 4). *P < 0.05, **P < 0.01, ***P < 0.001, ****P < 0.0001. Data were expressed as mean ± standard error

We then validated the expression of several hub genes in a rat model of pilocarpine‐induced SE at 2d, 7d, and 35d after induction. Most of tested hub genes, including *C1qb, Stat3, Timp1, Lyz2, and Tlr2,* were up‐regulated significantly at 48h. The mRNA expression of *Serpine1* was up‐regulated at 35d. *C1qa* and *Tyrobp* were up‐regulated significantly in all three time points (Figure 6). The mRNA levels of a latent stage hub gene, *Cd68,* significantly increased in 2d and 7d. The mRNA level of a chronic stage hub gene *C3* increased remarkably 7d and 35d after SE induction.

## 4 DISCUSSION

Epileptogenesis in TLE is an intricate and heterogeneous process with different pathological mechanisms such as inflammation, neurodegeneration, BBB disruption, and synapse reorganization.^2^ To better understand the molecular mechanisms underlying epileptogenesis, it is necessary to integrate the results from various animal TLE models. To the best of our knowledge, our study is the first to systematically integrate the existing microarray analyses in rodent TLE models using RRA and to analyze gene expression patterns in different phases after SE induction. The advantages of RRA analysis include strong robustness to noise, ability to handle incomplete lists, assigning p‐value to each gene in the final list, and high computing efficiency.^10^ These advantages make RRA an excellent tool for microarray integration. We also identified the hub genes in each stage, and some of them were associated with seizure frequency or hippocampus sclerosis in TLE patients. Interestingly, we found that the hub genes in the latent phase, the most critical phase for epileptogenesis, were closely associated with the activation and phagocytic activity of microglia/macrophages, suggesting that microglia/macrophages may play crucial roles in epileptogenesis.^17^

The robust DEGs obtained by RRA analysis were significantly enriched in inflammatory response in all three phases of epileptogenesis, especially in the acute and latent stage. This is consistent with the previous results in Gorter's microarray analysis and Walker's proteomic analysis.^18^, ^19^ Inflammation could participate in epilepsy through increasing the BBB permeability, regulating neurotransmitter transport, promoting neuron degeneration, and influencing neurogenesis.^2^, ^20^ Some promising antiinflammatory small molecules have been evaluated for their capacities to fight against epilepsy and epileptogenesis.^21^, ^22^ Our RRA analysis further supports the importance of inflammatory response in epileptogenesis of TLE.

In our study, the acute stage is defined as the first 3 days after SE induction.^23^ The enriched biological processes in acute stage were found to be involved in inflammatory response, neuron apoptosis, angiogenesis, and leukocyte migration, which are important pathological changes in the acute stage and may affect the severity of seizure.^2^, ^24^ The six hub genes identified in the acute phase have also been reported in previous microarrays studies.^8^ Specifically, we found *Tlr2, Stat3, and Lgals3* were positively correlated with seizure frequency. In addition, *Lgals3* and *Serpine1* were also associated with HS, further strengthening the importance of these hub genes. *Tlr2*, *Stat3* and *Ptgs2* are well‐known mediators of neuroinflammation and are potential antiseizure or antiepilepsy targets.^21^, ^25^, ^26^, ^27^ CD44 is an extracellular adhesion molecule expressed by glial cells and neurons. It regulates neurite outgrowth, leukocyte homing, synaptic transmission, and BBB integrity.^28^ It is reported that CD44 expression coincided with early mossy fiber sprouting at 3 days following pilocarpine‐induced SE.^29^ Our qRT‐PCR results showed that the hub gene *Timp1* was strongly up‐regulated in the acute stage. TIMP1, as a metalloproteinases inhibitor, may affect BBB integrity through inhibiting the activity of matrix metalloproteinases‐9 (MMP‐9).^30^ TIMP1 may also inhibit neuron apoptosis independent of MMP‐9.^31^ Jourquin et al found that TIMP‐1 deficiency had no influence on MMP‐9 activity and kainate‐induced seizure. However, TIMP‐1–deficient mice became resistant to excitotoxicity and exhibited reduced mossy fiber sprouting in kainate‐induced seizure.^32^ SERPINE1, a member of serine protease inhibitor family, plays roles in regulating microglial cell adhesion and phagocytosis, cell migration, glioblastoma cell adhesion, and fibrinogen deposition in ischemic stroke.^33^, ^34^, ^35^ SERPINE1 may protect neurons against N‐methyl‐D‐aspartate receptor–mediated excitotoxicity.^36^ More studies should be conducted to further examine the function of CD44, TIMP1, and SERPINE1 in epilepsy.

The latent stage is defined as a critical period when an epileptogenic neural network progressively matures and culminates in the appearance of SRS. The average length of latent period, starting from the initial injury to the first SRS, is 14 days.^23^ Our analysis showed that the changes in gene expression profile and protein interaction network were complicated in the latent stage, reflecting high molecular activities. The RRA DEGs in the latent stage were mostly enriched in neuroinflammatory response, regulation of cytokine production, and integrin‐mediated signaling pathway, which might associate with neural circuit reorganization in epileptogenesis.^2^ Surprisingly, the 5 hub genes identified in the latent stage were all related to microglia/macrophage activation and phagocytosis. *Aif1* encodes IBA1, an actin‐binding protein, and a popular cell‐type marker for microglia/macrophages. IBA1 expression is highly up‐regulated in microglia and macrophages after CNS injuries.^37^, ^38^ *Cd68* encodes CD68, a transmembrane glycoprotein highly expressed by the monocyte lineage cells, including circulating and tissue macrophages.^39^ *Emr1* encodes F4/80, a cell‐type marker for microglia/macrophages. *Tyrobp* encodes an adapter protein that transmits the signals from Trem2 and CR3 activation, and appears to be critical in microglial phagocytosis.^40^ *Lyz2* encodes a lysosome protein that is related to macrophage phagocytosis.^41^ The mRNA level of *Cd68 and Tyrobp* was also significantly up‐regulated in the latent stage after epilepsy in the current study. These results suggest that the activation and phagocytic activity of microglia/macrophage could play a critical role in epileptogenesis of TLE. Indeed, activated microglia has been detected in brain tissues of both TLE animals and TLE patients.^42^, ^43^ Microglia and macrophages exert pleiotropic roles in epilepsy and other forms of CNS injuries.^44^, ^45^, ^46^, ^47^ Activated microglia/macrophages produce M1 proinflammatory factors to exacerbate seizure or M2 antiinflammatory factors to promote tissue repair.^20^, ^48^ Microglia also regulates neuronal network excitability and synapse reorganization by secreting neurotrophic factors.^48^ In addition, microglia could prevent abnormal neurons from incorporating into dentate neural circuit by eliminating newborn or apoptotic cells in the epileptic hippocampus.^49^ Like in other nervous system diseases,^50^, ^51^ microglia may participate in the neural circuit remodeling through synapse pruning in epilepsy. Future work is warranted to elucidate the molecular mechanisms for microglia functions and identify targets to adjust proepileptogenic/antiepileptogenic microglia/macrophage responses.

The chronic period was the time when SRS occurs and epileptic neural network matures.^52^ Our analysis showed that inflammation still played important roles in chronic stage. Hub genes identified in the chronic stage, including *C1qa, C1qb, C3, Tyrobp* and *Lgals3,* are also robustly up‐regulated in the latent stage (*C1qa, C1qb, C3,* and *Tyrobp*) or in all three stages (*Lgals3*). Importantly, *Lgals3* was associated with seizure frequency and HS in TLE. All of these results supported a sustained role of epileptogenic genes in the chronic phase. Although the exact mechanisms how C1qa, C1qb, and C3 contribute to epileptogenesis remain unclear, some studies suggest that they may work in concert with activated microglia to enhance synapse pruning.^2^, ^53^ Another key gene in chronic stage, *Tyrobp*, acts as a signaling adaptor protein for TREM2 and CR3 in dendritic cells, macrophages and microglia. Recent studies revealed a critical role of TREM2/TYROBP signaling in the regulation of microglial phagocytosis in Alzheimer's disease and other neurological diseases.^54^, ^55^ The role of TYROBP in epilepsy is still unknown and deserves further exploration. LGALS3 is abundantly expressed and secreted by activated microglia and acts as an endogenous TLR4 ligand to promote microglial activation.^56^ Despite a strong up‐regulation in microglial cells, mice lacking LGALS3 only exhibited limited neuroprotection in cerebral cortex and no effect in hippocampus 3 days following pilocarpine injection.^57^ The role of LGALS3 in epilepsy needs to be further determined.

Our study has several limitations. First, the microarray detects the alterations in transcriptional level but not protein level. Proteomics research should be performed to confirm our conclusions. Second, due to limited amount of high‐throughput RNAseq data in epileptic animal models in GEO database, we only analyzed the microarray data which are not as accurate in detecting gene expression as high‐throughput sequencing. Third, we integrated both the rat and mouse microarrays to obtain the genes robustly expressed across different species. However, different species may respond to SE differently, which may confound our data interpretation. In addition, a cutoff of |log_2_ FC|> 0.5 was used to screen robust DEGs in RRA analysis to avoid missing important genes. A loose cutoff, however, could also bring several less significant genes.

In summary, using RRA analysis, we found that the robust DEGs were enriched in inflammation response in all three phases. We also identified the hub genes in each stage of epileptogenesis. Some hub genes were associated with seizure frequency or hippocampus sclerosis. Our results provide novel insights into the molecular mechanisms of TLE and highlight the importance of microglia/macrophage responses and neuroinflammation in epileptogenesis.

## DISCLOSURE

None.

## Supporting information

Supplementary Material

## Data Availability

The data that support the findings of this study are available from the corresponding author upon reasonable request.
