# Supplementary material for: Bioinformatic analysis identifies key transcriptome signatures in temporal lobe epilepsy
Source: CNS Neurosci Ther. 2020 Nov 22;26(12):1266–77. doi: 10.1111/cns.13470 (PMC7702228; doi:10.1111/cns.13470)
Supplement: Supplementary file 1 — Supplementary Material [file CNS-26-1266-s001.docx]

Figure S1: Boxplots of the six microarray datasets after standardization by RMA (Robust Multi-Array Average) algorithm. (A) [GSE14763](https://www.ncbi.nlm.nih.gov/geo/query/acc.cgi?acc=GSE63067), (B) [GSE47752](https://www.ncbi.nlm.nih.gov/geo/query/acc.cgi?acc=GSE48452),, (C) [GSE49030](https://www.ncbi.nlm.nih.gov/geo/query/acc.cgi?acc=GSE66676), (D)[GSE49849](https://www.ncbi.nlm.nih.gov/geo/query/acc.cgi?acc=GSE72756), (E) [GSE73878](https://www.ncbi.nlm.nih.gov/geo/query/acc.cgi?acc=GSE89632), (F) [GSE88992](https://www.ncbi.nlm.nih.gov/geo/query/acc.cgi?acc=GSE107231). Each box in boxplot represents the log2 intensity distribution of all genes in one sample of microarray. The horizontal line in the inner of the box represents the median line of log2 intensity and the upside and downside of the box represented the quartile value. The white box represented the control group. The red box, yellow box and the blue box represented the acute, latent and chronic samples in the microarrays respectively.

**
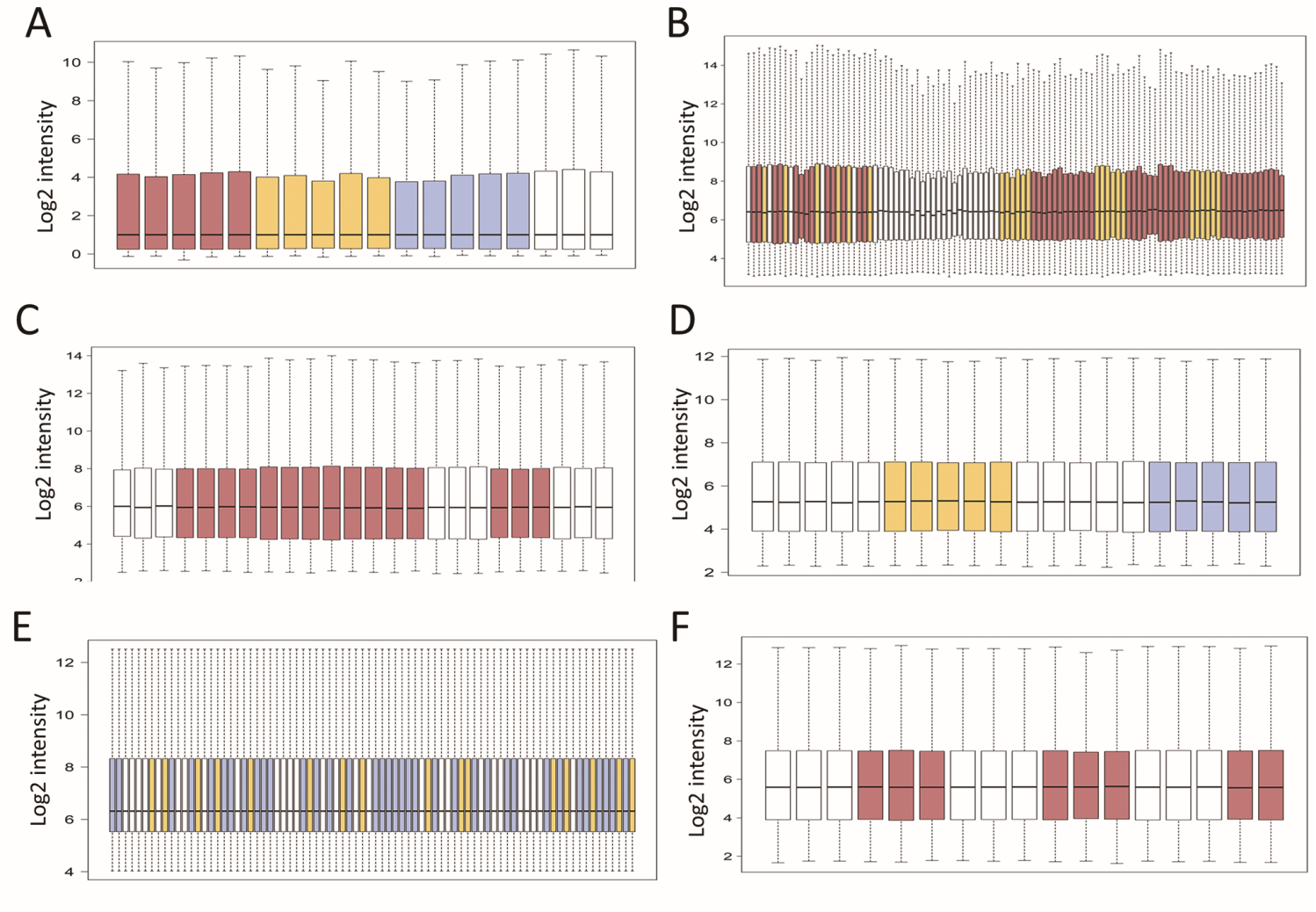
**

Table S1: List of rat primer used in our study.

| Primer | Sequence |
| --- | --- |
| C1QA | Forward: CACGGAGGCAGGAACATCAT; Reverse: GGCTCCCCTCTCTCTCCTTT |
| C3 | Forward: GCGGTACTACCAGACCATCG; Reverse: CTTCTGGCACGACCTTCAGT |
| TIMP-1 | Forward: CCAGCGTTATGAGATCAAGA; Reverse: CAGGCAGTGATGTGCAAATT |
| TYROBP | Forward: GTGACAATTACCCAGGATGCGA; Reverse: CTGTTTCCGGGTCCCGTCTG |
| C1QB | Forward: GACGTTTTTGGGAGGGGACA; Reverse: GGGCCTCCTGTGTATGGAATC |
| TLR2 | Forward: TGGAGGTCTCCAGGTCAAATCT; Reverse: TGTTTGCTGTGAGTCCCGAG |
| CD68 | Forward: TGTTCAGCTCCAAGCCCAAA; Reverse: GCTCTGATGTCGGTCCTGTTT |
| STAT3 | Forward: AAGCTGACCCAGGTAGTGCT; Reverse: TCCATGTCAAACGTGAGCGA |
| GAPDH | Forward: ATGACTCTACCCACGGCAAG; Reverse: TACTCAGCACCAGCATCACC |
| LYZ2 | Forward: AAGATCTATGAACGCTGTGAGT; Reverse: TGAGCTAAACACACCCAGTCT |
| SERPINE1 | Forward: CGTCTTCCTCCACAGCCATT; Reverse：GCTGGCCCATGAAGAGGATT |

Table S2: The robust DEGs identified by RRA method in acute, latent and chronic stages of epileptogenesis. The robust differentially expressed genes (DEGs) obtained by integrating multiple microarrays using robust rank aggregation (RRA) algorithm in three stages of epilepsy. RRA algorithm assigned P value and log2 |Fold Change (FC)| to each gene and ranked the genes by the assigned P value. Genes with Bonferroni adjusted P value < 0.05 and log2 FC > 0.5 were strictly kept in the final aggregated lists as the robust DEGs.

| upregulated gene | | | | | | | |
| --- | --- | --- | --- | --- | --- | --- | --- |
| acute stage | | latent stage | | latent stage | | chronic stage | |
| gene symbol | Adj.pvalue | gene symbol | Adj.pvalue | gene symbol | Adj.pvalue | gene symbol | Adj.pvalue |
| Hspb1 | 2.29E-07 | Lgals3 | 4.78E-07 | Ptpn6 | 0.01678 | Serping1 | 0.000196 |
| Lcn2 | 3.44E-06 | Serping1 | 2.26E-05 | Abi3 | 0.016864 | Timp1 | 0.000196 |
| Timp1 | 2.02E-05 | Gpnmb | 2.45E-05 | Grn | 0.017963 | Gpnmb | 0.00081 |
| Spp1 | 2.20E-05 | Timp1 | 2.76E-05 | Adgre1 | 0.01939 | Ly86 | 0.007644 |
| Cd14 | 8.49E-05 | Cd74 | 6.64E-05 | Ctss | 0.019837 | Pld4 | 0.009879 |
| Cd44 | 0.000188 | Gpr84 | 0.000119 | Cmtm7 | 0.021703 | Cd74 | 0.010373 |
| Lgals3 | 0.000324 | Gfap | 0.00018 | Ifitm3 | 0.023186 | Nptx2 | 0.012231 |
| S100a10 | 0.00054 | Arl11 | 0.000218 | Igf1 | 0.023976 | Lgals3 | 0.013091 |
| Gpr84 | 0.00055 | Laptm5 | 0.000245 | Plin2 | 0.024391 | C3 | 0.013795 |
| Cd68 | 0.000561 | Ly86 | 0.000262 | C1qc | 0.025472 | Gfap | 0.017997 |
| Vim | 0.000648 | Cd53 | 0.000506 | Tlr7 | 0.030332 | C1qa | 0.019871 |
| Gadd45a | 0.000783 | Nckap1l | 0.000535 | Lat2 | 0.031198 | Tyrobp | 0.029661 |
| Plek | 0.001281 | Hspb1 | 0.000566 | Rab32 | 0.031561 | Hspb1 | 0.033343 |
| Serpine1 | 0.001486 | Clec7a | 0.000631 | Tmem176b | 0.031989 | C1qb | 0.03501 |
| Tubb6 | 0.001958 | Plek | 0.000666 | Enpp6 | 0.035339 | Cxcl16 | 0.040966 |
| Msn | 0.002092 | C1qa | 0.000778 | Cd63 | 0.036275 |  |  |
| Blnk | 0.003811 | C3 | 0.001094 | S100a3 | 0.038723 |  |  |
| Maff | 0.004503 | Cd37 | 0.001524 | Pik3ap1 | 0.04072 |  |  |
| Adamts1 | 0.004962 | Vim | 0.001986 | Evi2b | 0.041829 |  |  |
| Tnfrsf12a | 0.005982 | Cd68 | 0.002029 | Npc2 | 0.043359 |  |  |
| Cdkn1a | 0.006166 | Cd180 | 0.002067 | Casp1 | 0.046124 |  |  |
| Ch25h | 0.006355 | Isg15 | 0.002481 |  |  |  |  |
| Clic1 | 0.006745 | Apobec1 | 0.003492 |  |  |  |  |
| Ccl3 | 0.007196 | Tyrobp | 0.003533 |  |  |  |  |
| Lrg1 | 0.008252 | Lsp1 | 0.004287 |  |  |  |  |
| Atf3 | 0.00855 | Pld4 | 0.004479 |  |  |  |  |
| Fosb | 0.00855 | Aif1 | 0.004481 |  |  |  |  |
| Capg | 0.009474 | Mvp | 0.00453 |  |  |  |  |
| Sprr1a | 0.009822 | Trh | 0.004976 |  |  |  |  |
| Tnfrsf1a | 0.011212 | Ch25h | 0.006867 |  |  |  |  |
| S1pr3 | 0.014783 | Unc93b1 | 0.007718 |  |  |  |  |
| Nupr1 | 0.015515 | Lyz2 | 0.007867 |  |  |  |  |
| Tlr2 | 0.016145 | Fes | 0.008094 |  |  |  |  |

(Table S2) coutinued

| upregulated gene | | | | | | | |  |  |  |  |  |  |  |
| --- | --- | --- | --- | --- | --- | --- | --- | --- | --- | --- | --- | --- | --- | --- |
| acute stage | | latent stage | | latent stage | | chronic stage | |  |  |  |  |  |  |  |
| gene symbol | Adj.pvalue | gene symbol | Adj.pvalue | gene symbol | Adj.pvalue | gene symbol | Adj.pvalue |  |  |  |  |  |  |  |
| Serpina3n | 0.016273 | C1qb | 0.008726 |  |  |  |  |  |  |  |  |  |  |  |
| Ptgs2 | 0.016472 | Ccl3 | 0.009223 |  |  |  |  |  |  |  |  |  |  |  |
| Ifitm3 | 0.017872 | Vav1 | 0.009308 |  |  |  |  |  |  |  |  |  |  |  |
| Tgm2 | 0.019147 | Rsad2 | 0.010466 |  |  |  |  |  |  |  |  |  |  |  |
| Cd9 | 0.020489 | Msn | 0.010939 |  |  |  |  |  |  |  |  |  |  |  |
| Itgb2 | 0.020952 | Myo1f | 0.011036 |  |  |  |  |  |  |  |  |  |  |  |
| Stat3 | 0.023215 | Gbp2 | 0.011133 |  |  |  |  |  |  |  |  |  |  |  |
| Zfp36 | 0.029272 | Cd86 | 0.011231 |  |  |  |  |  |  |  |  |  |  |  |
| Gbp2 | 0.030489 | Oasl2 | 0.012096 |  |  |  |  |  |  |  |  |  |  |  |
| Bag3 | 0.041908 | Nupr1 | 0.014714 |  |  |  |  |  |  |  |  |  |  |  |
| Pdlim4 | 0.044039 | S100a10 | 0.015324 |  |  |  |  |  |  |  |  |  |  |  |
| Inhba | 0.045412 | Lox | 0.015448 |  |  |  |  |  |  |  |  |  |  |  |
| downregulated gene | | | | | | | |  |  |  |  |  |  |  |
| acute stage |  | latent stage | | chronic stage | |  |  |  |  |  |  |  |  |  |
| gene symbol | Adj.Pvalue | Name | Adj.Pvalue | Name | Adj.Pvalue |  |  |  |  |  |  |  |  |  |
| Htr5b | 5.00E-06 | Gdf10 | 9.57E-07 | Gdf10 | 2.79E-06 |  |  |  |  |  |  |  |  |  |
| Plk5 | 0.00022754 | Htr5b | 0.00020427 | Ar | 0.01525135 |  |  |  |  |  |  |  |  |  |
| Lin7b | 0.00038929 | Gpc3 | 0.00244971 |  |  |  |  |  |  |  |  |  |  |  |
| Lzts3 | 0.00089439 | Necab2 | 0.01520007 |  |  |  |  |  |  |  |  |  |  |  |
| Gdf10 | | | | | | | | 0.00185461 | Trhr | 0.01983975 |  |  |  |  |
| Slc26a10 | | 0.00372791 | | Cxcl12 | | 0.02439141 |  |  |  |  |  |  |  |  |
| Arhgap20 | 0.00376783 | Slc6a1 | 0.03580498 |  |  |  |  |  |  |  |  |  |  |  |
| Lct | 0.00380037 | Fndc5 | 0.03894555 |  |  |  |  |  |  |  |  |  |  |  |
| Ntf3 | 0.0064186 | Etv1 | 0.04176055 |  |  |  |  |  |  |  |  |  |  |  |
| Camkk2 | 0.0064186 |  |  |  |  |  |  |  |  |  |  |  |  |  |
| Dsp | 0.00855039 |  |  |  |  |  |  |  |  |  |  |  |  |  |
| Pcdh19 | 0.01502415 |  |  |  |  |  |  |  |  |  |  |  |  |  |
| Kctd4 | 0.01536319 |  |  |  |  |  |  |  |  |  |  |  |  |  |
| Rab26 | 0.02529629 |  |  |  |  |  |  |  |  |  |  |  |  |  |
| Frzb | 0.03303778 |  |  |  |  |  |  |  |  |  |  |  |  |  |

Table S3: The pathway and process enrichment analysis identified by Metascape of RRA genes in acute, latent and chronic stages. In Metascape, pathway and process enrichment analysis has been carried out with the following ontology sources: KEGG Pathway, GO Biological Processes, Reactome Gene Sets, Canonical Pathways, CORUM, TRRUST, DisGeNET and PaGenBase. Terms with a p-value < 0.01, a minimum count of 3, and an enrichment factor > 1.5 are collected and grouped into clusters based on their membership similarities. A similarity > 0.3 are considered a cluster. Log p is the p-value in log base 10.

| GO | Description | LogP_Acute | LogP_Chronic | LogP_Latent |
| --- | --- | --- | --- | --- |
| GO:0048145 | regulation of fibroblast proliferation | -2.40388 | 0 | -2.13348 |
| GO:2000045 | regulation of G1/S transition of mitotic cell cycle | -2.2955 | 0 | -2.02743 |
| GO:0031329 | regulation of cellular catabolic process | -2.9185 | 0 | -2.30043 |
| mmu04620 | Toll-like receptor signaling pathway | -3.67258 | 0 | -2.22658 |
| GO:0010563 | negative regulation of phosphorus metabolic process | -3.62884 | 0 | -2.33075 |
| GO:0043086 | negative regulation of catalytic activity | -3.65271 | 0 | -2.33662 |
| GO:0071396 | cellular response to lipid | -4.05493 | 0 | -3.99659 |
| GO:0045637 | regulation of myeloid cell differentiation | -2.37912 | 0 | -6.90085 |
| GO:0150076 | neuroinflammatory response | -2.94776 | 0 | -9.7736 |
| R-MMU-6798695 | Neutrophil degranulation | -3.90598 | 0 | -7.36708 |
| GO:0007229 | integrin-mediated signaling pathway | -2.60255 | 0 | -5.96075 |
| GO:0072676 | lymphocyte migration | -2.4623 | 0 | -4.41621 |
| R-MMU-168898 | Toll-like Receptor Cascades | -2.15972 | 0 | -3.89934 |
| R-MMU-1433557 | Signaling by SCF-KIT | 0 | 0 | -3.42583 |
| GO:0051590 | positive regulation of neurotransmitter transport | 0 | 0 | -3.04837 |
| GO:0000768 | syncytium formation by plasma membrane fusion | 0 | 0 | -3.92753 |
| R-MMU-1643685 | Disease | 0 | 0 | -2.53264 |
| GO:0031214 | biomineral tissue development | 0 | 0 | -2.50079 |
| GO:0018108 | peptidyl-tyrosine phosphorylation | 0 | 0 | -2.90046 |
| GO:0048771 | tissue remodeling | 0 | 0 | -2.20209 |
| R-MMU-1679131 | Trafficking and processing of endosomal TLR | 0 | 0 | -4.97948 |
| mmu04142 | Lysosome | 0 | 0 | -4.01178 |
| GO:0002275 | myeloid cell activation involved in immune response | 0 | 0 | -4.77493 |
| R-MMU-983695 | Antigen activates B Cell Receptor (BCR) leading to generation of second messengers | 0 | 0 | -4.08642 |
| GO:0014002 | astrocyte development | 0 | 0 | -4.98083 |

Table S3 continued

| GO | Description | LogP_Acute | LogP_Chronic | LogP_Latent |
| --- | --- | --- | --- | --- |
| GO:0002920 | regulation of humoral immune response | 0 | 0 | -5.0293 |
| GO:0051607 | defense response to virus | 0 | 0 | -6.72163 |
| GO:0034154 | toll-like receptor 7 signaling pathway | 0 | 0 | -7.66618 |
| GO:0001906 | cell killing | 0 | -3.22185 | -3.983 |
| GO:0070372 | regulation of ERK1 and ERK2 cascade | 0 | -2.66613 | -3.78089 |
| GO:0002697 | regulation of immune effector process | 0 | -3.42801 | -5.41783 |
| GO:1901214 | regulation of neuron death | 0 | -2.44858 | -2.56842 |
| GO:0002683 | negative regulation of immune system process | 0 | -4.43699 | -9.65469 |
| GO:0002253 | activation of immune response | 0 | -4.23658 | -8.10052 |
| GO:0031667 | response to nutrient levels | -3.09613 | 0 | 0 |
| GO:0006606 | protein import into nucleus | -3.89073 | 0 | 0 |
| GO:0071363 | cellular response to growth factor stimulus | -3.38462 | 0 | 0 |
| GO:0045646 | regulation of erythrocyte differentiation | -3.3834 | 0 | 0 |
| GO:0032970 | regulation of actin filament-based process | -3.6986 | 0 | 0 |
| GO:0034113 | heterotypic cell-cell adhesion | -3.25883 | 0 | 0 |
| mmu04066 | HIF-1 signaling pathway | -3.54323 | 0 | 0 |
| GO:0010677 | negative regulation of cellular carbohydrate metabolic process | -3.35744 | 0 | 0 |
| GO:0030728 | ovulation | -4.2651 | 0 | 0 |
| GO:0097191 | extrinsic apoptotic signaling pathway | -5.27527 | 0 | 0 |
| GO:0008544 | epidermis development | -4.4407 | 0 | 0 |
| GO:2000377 | regulation of reactive oxygen species metabolic process | -4.50962 | 0 | 0 |
| GO:0055094 | response to lipoprotein particle | -4.2651 | 0 | 0 |
| GO:0030855 | epithelial cell differentiation | -5.83179 | 0 | 0 |
| mmu04657 | IL-17 signaling pathway | -2.60255 | 0 | 0 |
| mmu04640 | Hematopoietic cell lineage | -2.54953 | 0 | 0 |
| GO:0034612 | response to tumor necrosis factor | -2.65608 | 0 | 0 |
| GO:0009725 | response to hormone | -2.27639 | 0 | 0 |
| GO:0072657 | protein localization to membrane | -2.22134 | 0 | 0 |
| GO:0038034 | signal transduction in absence of ligand | -2.7624 | 0 | 0 |
| GO:0022604 | regulation of cell morphogenesis | -2.98376 | 0 | 0 |
| GO:0060395 | SMAD protein signal transduction | -2.56255 | 0 | 0 |
| GO:0045596 | negative regulation of cell differentiation | -2.04333 | 0 | 0 |
| R-MMU-109581 | Apoptosis | -2.56255 | 0 | 0 |
| GO:0045600 | positive regulation of fat cell differentiation | -2.98483 | 0 | 0 |
| GO:0045765 | regulation of angiogenesis | -8.7676 | -2.67796 | 0 |
| GO:0050900 | leukocyte migration | -6.12469 | -2.55401 | -5.29287 |
| GO:0008285 | negative regulation of cell proliferation | -6.14401 | -2.64671 | -4.33786 |
| GO:0097190 | apoptotic signaling pathway | -7.67191 | -3.94821 | -2.12911 |
| GO:0043900 | regulation of multi-organism process | -2.18616 | -3.53997 | -6.62017 |
| GO:0006897 | endocytosis | -3.48091 | -3.54025 | -4.74459 |
| GO:0009611 | response to wounding | -4.67957 | -4.27982 | -4.67285 |

Table S3 continued

| GO | Description | LogP_Acute | LogP_Chronic | LogP_Latent |
| --- | --- | --- | --- | --- |
| GO:0001817 | regulation of cytokine production | -4.39525 | -4.79465 | -8.26901 |
| GO:0006954 | inflammatory response | -9.75975 | -6.02271 | -10.0675 |

Table S4: The protein-protein interaction network of RRA genes in acute, latent and chronic stages identified by STRING database. In the PPI network, each node represents a protein encoded by DEGs and the edge between nodes represents the interaction of the molecules. Count represented the number of edges of protein encoded by RRA genes.

| Acute stage | count | Latent stage | count | Latent stage | count | Chronic stage | count |
| --- | --- | --- | --- | --- | --- | --- | --- |
| TLR2 | 18 | CD68 | 31 | OASL2 | 5 | C1QA | 9 |
| STAT3 | 17 | EMR1 | 27 | S100A10 | 5 | C1QB | 9 |
| TIMP1 | 17 | TYROBP | 27 | PIK3AP1 | 4 | TYROBP | 8 |
| CD44 | 16 | LYZ2 | 25 | VIM | 4 | LGALS3 | 6 |
| PTGS2 | 14 | AIF1 | 24 | CD180 | 3 | C3 | 5 |
| SERPINE1 | 14 | C1QA | 23 | GPC3 | 3 | CD74 | 5 |
| CCL3 | 13 | C1QB | 23 | GPNMB | 3 | CXCL16 | 5 |
| LGALS3 | 13 | CTSS | 23 | HSPB1 | 3 | LY86 | 5 |
| ATF3 | 12 | LAPTM5 | 21 | MSN | 3 | PLD4 | 5 |
| CD68 | 12 | LY86 | 21 | CH25H | 2 | SERPING1 | 4 |
| SPP1 | 12 | NCKAP1L | 21 | LAT2 | 2 | GFAP | 3 |
| CD14 | 11 | TLR7 | 21 | TRH | 2 | HSPB1 | 3 |
| ITGB2 | 9 | C1QC | 20 | ABI3 | 1 | TIMP1 | 3 |
| LCN2 | 9 | CD53 | 20 | ARL11 | 1 | GPNMB | 2 |
| TNFRSF1A | 8 | PLD4 | 20 | ETV1 | 1 | AR | 1 |
| CDKN1A | 6 | PLEK | 20 | EVI2B | 1 | GDF10 | 1 |
| PLEK | 6 | CD86 | 19 | FNDC5 | 1 |  |  |
| HSPB1 | 5 | PTPN6 | 18 | GDF10 | 1 |  |  |
| MSN | 5 | VAV1 | 18 | GPR84 | 1 |  |  |
| VIM | 5 | CASP1 | 16 | S100A3 | 1 |  |  |
| IFITM3 | 4 | MYO1F | 16 | SLC6A1 | 1 |  |  |
| SERPINA3N | 4 | CD74 | 15 | TRHR | 1 |  |  |
| TNFRSF12A | 4 | LGALS3 | 15 |  |  |  |  |
| ADAMTS1 | 3 | UNC93B1 | 15 |  |  |  |  |
| CAPG | 3 | CCL3 | 14 |  |  |  |  |
| GADD45A | 3 | IGF1 | 13 |  |  |  |  |
| TGM2 | 3 | GFAP | 12 |  |  |  |  |
| ZFP36 | 3 | TIMP1 | 12 |  |  |  |  |
| BAG3 | 2 | C3 | 11 |  |  |  |  |
| CD9 | 2 | CLEC7A | 11 |  |  |  |  |
| CH25H | 2 | CXCL12 | 9 |  |  |  |  |
| FOSB | 2 | CD63 | 7 |  |  |  |  |
| LRG1 | 2 | LOX | 7 |  |  |  |  |
| MAFF | 2 | GRN | 6 |  |  |  |  |
| BLNK | 1 | ISG15 | 6 |  |  |  |  |
| GBP2 | 1 | RSAD2 | 6 |  |  |  |  |
| GDF10 | 1 | SERPING1 | 6 |  |  |  |  |
| GPR84 | 1 | CD37 | 5 |  |  |  |  |
| INHBA | 1 | GBP2 | 5 |  |  |  |  |
| S100A10 | 1 | IFITM3 | 5 |  |  |  |  |

Table S4 continued

| SPRR1A | 1 | NPC2 | 5 |  |  |  |  |
| --- | --- | --- | --- | --- | --- | --- | --- |
